# One-pot synthesis of a [c2]daisy-chain-containing hetero[4]rotaxane *via* a self-sorting strategy†
†Electronic supplementary information (ESI) available: Full experimental procedures and characterization data for all compounds. See DOI: 10.1039/c5sc04844c



**DOI:** 10.1039/c5sc04844c

**Published:** 2016-01-12

**Authors:** Xin Fu, Qi Zhang, Si-Jia Rao, Da-Hui Qu, He Tian

**Affiliations:** a Key Laboratory for Advanced Materials and Institute of Fine Chemicals , East China University of Science and Technology , 130 Meilong Road , Shanghai , 200237 , China . Email: dahui_qu@ecust.edu.cn

## Abstract

The construction and efficient synthesis of hetero[*n*]rotaxanes with high structural complexity are always attractive challenges. Herein, we demonstrate a facile one-pot preparation of a hetero[4]rotaxane, by employing a self-sorting strategy, which contains an interpenetrated dibenzo-24-crown-8 (**DB24C8**) based [c2]daisy chain structure and is ended with a benzo-21-crown-7 (**B21C7**) based rotaxane at each side. The key to the design involved encoding the selective threading using a steric hindrance-related “language”, where highly selective self-assemblies occurred in a three-component self-sorting process, which included the threading of a benzylalkylammonium into a **B21C7** and interpenetrated dimerized formation of a **DB24C8** based [c2]daisy chain simultaneously; the precise pre-assembled system resulted in the efficient synthesis of hetero[4]rotaxane with a high-level of structural complexity under the “CuAAC” reaction.

## Abstract 2

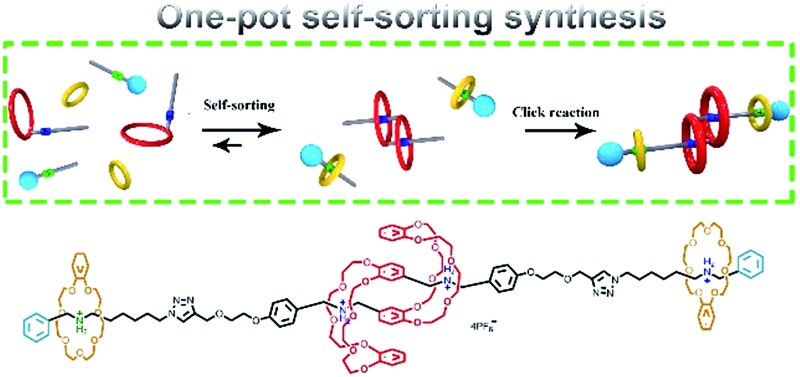
Here we demonstrated a facile and efficient one-pot synthesis of a [c2]daisy-chain-containing hetero[4]rotaxane *via* a self-sorting strategy.

## Introduction

In the last twenty years, a variety of mechanically interlocked molecules (MIMs), especially rotaxanes and catenanes, have been delicately designed and constructed.^1^ The unique structural features of MIMs are utilized by scientists to birth elegant functional molecules,^2^ indicating the great potential of MIMs in functional materials. As a modification platform for functional groups, the structural complexity of MIMs significantly provides infinite possibilities in designing novel functional molecular machines.^3^ Hence, the construction and efficient synthesis of MIMs with high structural complexity have been greatly attractive but challenging.^4^ Recently, much attention has been focused on the topological,^3*a*^ interpenetrating,^5^ knotted^6^ structures of MIMs with high structural complexity. In this intriguing family, [c2] daisy chain molecules,^5^ a class of rotaxanes constructed from the dimerization of AB-type linear monomers with two self-complementary units, A (host) and B (guest), have shown their talent in mimicking the contraction and extension movements of natural biological machines.^5*l*^ Various elegant [c2] daisy chain molecules have been reported based on different host–guest systems including versatile macrocycles, such as crown ethers,^5b,e,h^ cucurbit[*n*]uril,^5*i*^ cyclodextrins,^5a,c,d^ pillarenes,^5g,m,p^ and cyclophanes.^5*o*^

A variety of hetero[*n*]rotaxanes, especially those comprising two or more different types of ring moiety, have been designed and constructed,^7^ some even in a one-step strategy.^7b,d^ This aroused our interest to integrate muscle-like daisy chain structures with hetero[*n*]rotaxanes to breed a hetero[*n*]rotaxane with a novel structural topology. However, it is difficult to synthesize this novel hetero[*n*]rotaxane based on traditional strategies for rotaxane synthesis,^8^ which are efficient in the preparation of typical [2]rotaxane but complicated when there is more than one type of ring moiety, due to them being “non-selective”. The key challenge is the dramatically increased complexity of the interpenetration process in the formation of a [c2]daisy chain when additional recognition sites and rings are incorporated into the multi-component self-assembling system. Hence, a programming language for highly selective self-assembly is desperately needed to integrate hetero[*n*]rotaxane with the daisy chain structure.

Self-sorting strategies have the unique capability of selective self-assembly in complex supramolecular systems.^9^ Schalley and co-workers^10^ have developed an efficient integrative self-sorting strategy for selective self-assembly to simultaneously incorporate two kinds of polyether macrocycles into a single axle molecule comprising two kinds of secondary ammoniums which, as a result, form a cascade-stoppered hetero[3]rotaxane. This self-sorting strategy has been proved to be very efficient in constructing many hetero[*n*]rotaxanes with increased structural complexity.^11^ However, to the best of our knowledge, this self-sorting strategy has not been proved to be appropriate for a system simultaneously involving both threading and interpenetration, which are known for the formation of pseudorotaxanes and daisy chain structures, respectively.

**Scheme 1:**
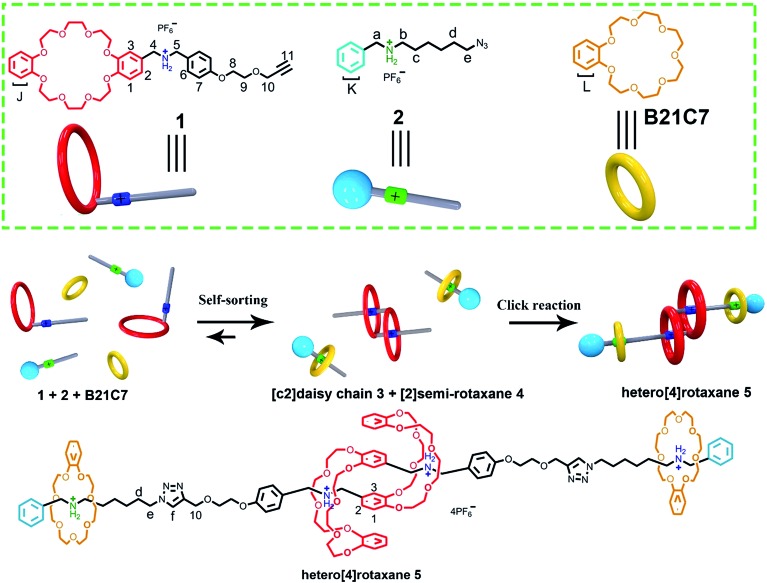
Schematic representation of the preparation of hetero[4]rotaxane **5** using a three-component modularized self-sorting system of **1**, **2** and **B21C7**. Only one main stereoisomer of hetero[4]rotaxane **5**, which is derived from the three possible [c2]daisy chain stereoisomers,^5*e*^ is shown here.

Herein, a facile one-pot synthesis of hetero[4]rotaxane **5** is successfully realized *via* a three-component (**1**, **2** and **B21C7** in Scheme 1) self-sorting strategy. In principle, these initial components (**1**, **2** and **B21C7** in Scheme 1) could assemble into several self-assembly species, while only two kinds of precursors, [c2]daisy chain **3** and [2]semi-rotaxane **4**, were successfully formed through the highly selective self-assembly process as determined by ^1^H NMR studies. In this pre-assembly process, both the self-interpenetration of compound **1** to form [c2]daisy chain **3** and the threading of **2** into the cavity of **B21C7** to form [2]semi-rotaxane **4** occurred simultaneously. Then the highly symmetrical hetero[4]rotaxane **5** containing [c2]daisy chain could be obtained by the following facile one-pot CuAAC click reaction.^8*d*^ We believe that the facile one-pot synthesis of hetero[4]rotaxane **5** would inspire the successful construction of MIMs with fascinating structures and potential functions.

## Results and discussion

The rational design of block building is a prerequisite to realizing the precise construction of a multi-component system. As shown in Scheme 1, initial substrates include compound **1** (the synthesis information from compounds **7** and **8** can be found in the ESI†), which contains a **DB24C8** macrocycle modified with a secondary dibenzylammonium branched chain; a secondary benzylalkylammonium **2**; and **B21C7**. The key part of the design involves the dibenzylammonium recognition site in compound **1**, where the phenyl group encodes the secondary ammonium as a selective site that only can be included by the larger macrocyclic **DB24C8**, but not by **B21C7**.^7b,10,12^ Therefore, even in one system, **B21C7** would not affect the formation of the [c2]daisy chain **3**. Meanwhile, this phenyl group also makes the secondary ammonium in compound **1** a preferred recognition site for **DB24C8**, over that in compound **2**.^10^ Considering the precise 1 : 1 proportion of **DB24C8** and the secondary ammonium in compound **1**, compound **2** would not affect the formation of [c2]daisy chain **3** from monomer molecule **1**, which has been proved by the ^1^H NMR measurements (Fig. S1†). As a result, the self-assembly of the initial molecules was precisely encoded, where the self-sorting process included simultaneous threading and interpenetration, and then [2]semi-rotaxane **4** and [c2]daisy chain **3** were generated, respectively. Finally, in the presence of Cu(MeCN)_4_PF_6_, the subsequent CuAAC click reaction between [c2]daisy chain **3** and [2]semi-rotaxane **4** afforded the formation of hetero[4]rotaxane **5** in a one-pot strategy. In the obtained hetero[4]rotaxane **5**, two **B21C7** rings can be stopped by the outer phenyl groups, while the central [c2]daisy chain structures are cascade-stopped by the **B21C7** rings.

**Fig. 1:**
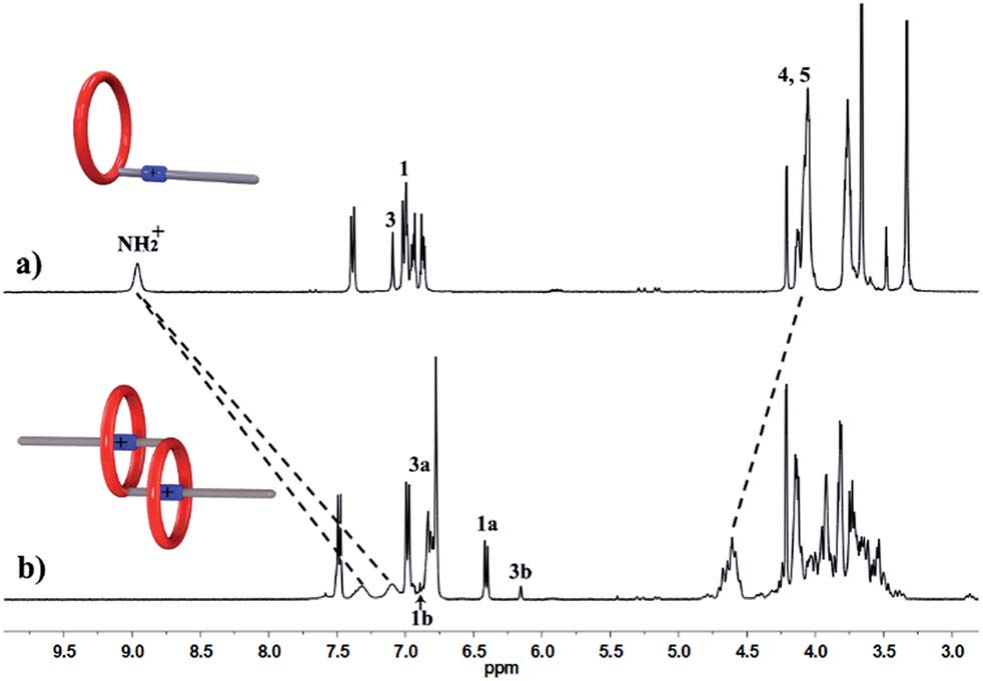
Partial ^1^H NMR spectra (400 MHz, 298 K) of compound **1** in (a) [D_6_]DMSO, and (b) [D_3_]acetonitrile.

**Fig. 2:**
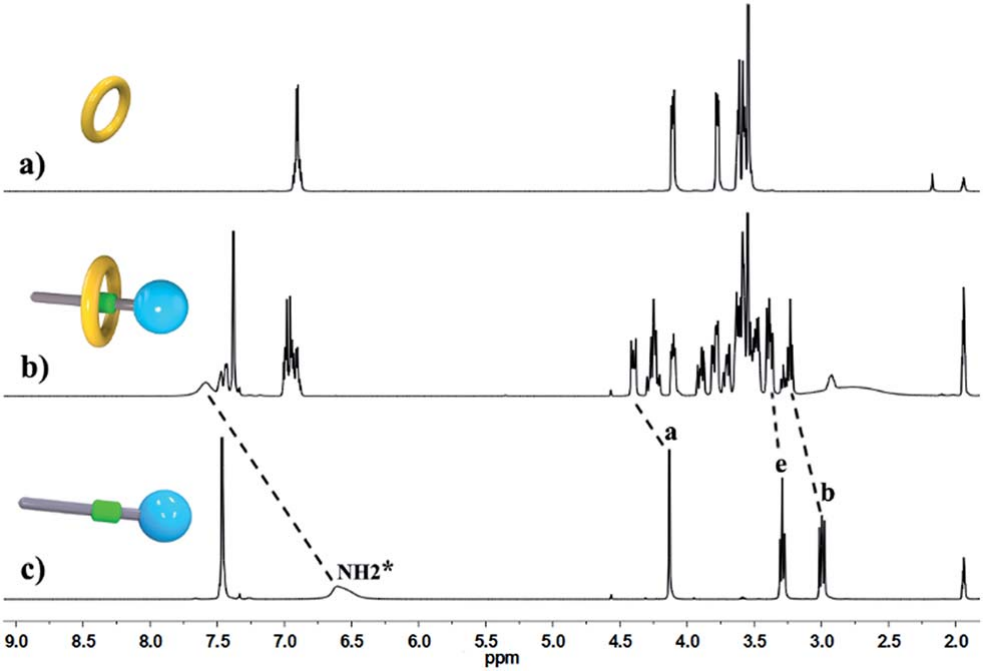
Partial ^1^H NMR spectra (400 MHz, 298 K, [D_3_]acetonitrile) of (a) **B21C7**, (b) a 1 : 1 mixture of **2** and **B21C7**, and (c) compound **2**.

**Fig. 3:**
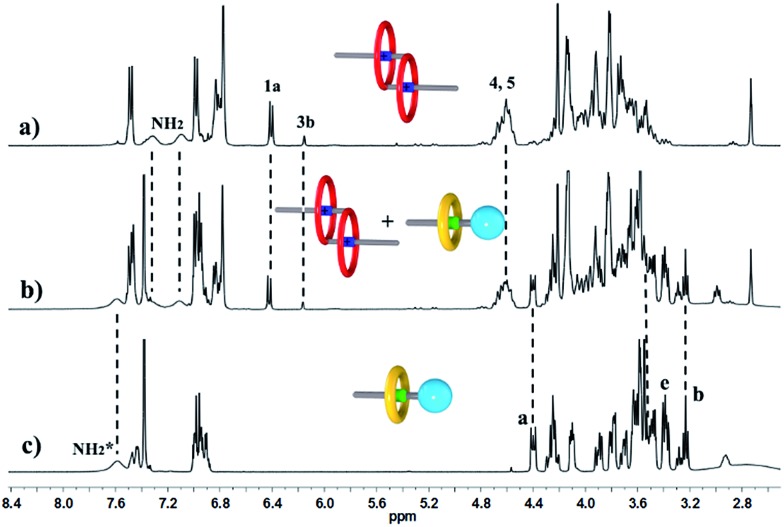
Partial ^1^H NMR spectra (400 MHz, 298 K, [D_3_]acetonitrile) of (a) compound **1**, (b) a 1 : 1 : 1 mixture of **1**, **2** and **B21C7**, and (c) a 1 : 1 mixture of **2** and **B21C7**.

We performed ^1^H NMR experiments to confirm the self-sorting behaviours in this three-component system, as shown in Fig. 1. The ^1^H NMR spectra revealed that **1** existed as a monomer in polar solvents, such as [D_6_]DMSO (Fig. 1a), with normal and simple ^1^H NMR signals. While in less polar solvents, such as [D_3_]acetonitrile (Fig. 1b), [c2]daisy chain **3** became the predominant species. The ^1^H NMR signals of the NH_2_^+^ protons and crown ether moiety shifted and split, becoming much broader and more complicated. As shown in Fig. 1b, two sets of signals for the NH_2_^+^ protons were observed at *δ* = 7.0–7.4 ppm, attributed to each ammonium facing the two non-symmetrical ends of the axle. According to the previous studies about the analogous [c2]daisy chains,^13,14^ there are three dimeric interlocked stereoisomers arising from the unsymmetrical substitution of the **DB24C8** ring, which were also observed in the ^1^H NMR spectrum of [c2]daisy chain **3** (Fig. 1b). As shown in Fig. 1, the aromatic protons of **DB24C8** also become split, especially protons H_1_ and H_3_. H_1_ splits into two sets of double peaks (H_1a_, 6.4 ppm and H_1b_, 6.9 ppm), and H_3_ splits into two sets of single peaks (H_3a_, 6.8 ppm and H_3b_, 6.2 ppm) with the ratio of 5 : 1, indicating the major supramolecular stereoisomer of the “meso” type, shown in Scheme 1, which is consistent with the previous report.^5*e*^ Meanwhile, the formation of [2]semi-rotaxane **4** from compound **2** and **B21C7** has been detected using the ^1^H NMR spectra shown in Fig. 2. After mixing compound **2** and **B21C7** in a 1 : 1 molar ratio, the signals of the NH_2_^+^ protons shifted upfield with a Δ*δ* of –0.97 ppm, and the signals of H_a_, H_b_ and H_e_ shifted downfield with a Δ*δ* of 0.26, 0.24 and 0.07 ppm, respectively, suggesting the formation of [2]semi-rotaxane **4**.^12^ Then, the three compounds **1**, **2** and **B21C7** were mixed in [D_3_]acetonitrile in a molar ratio of 1 : 1 : 1. ^1^H NMR spectra (Fig. 3) showed that the signals of H_1a_, H_3b_, H_4_ and H_5_ in [c2]daisy chain **3**, as well as those of H_a_, H_b_ and H_e_ in [2]semi-rotaxane **4**, remain constant. These observations indicate that the two pseudorotaxanes still exist as the predominant species in this three-component self-sorting system.

**Fig. 4:**
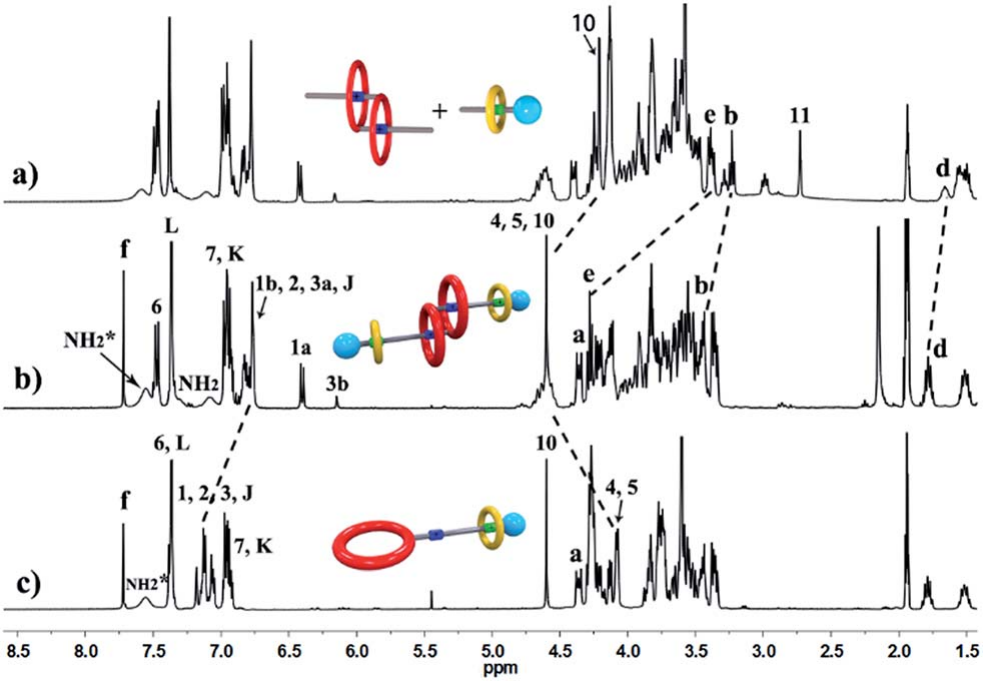
Partial ^1^H NMR spectra (400 MHz, 298 K, [D_3_]acetonitrile) of (a) a 1 : 1 : 1 mixture of **1**, **2** and **B21C7**, (b) hetero[4]rotaxane **5**, and (c) [2]rotaxane **6**.

The synthetic route for hetero[4]rotaxane **5** is outlined in Scheme 1. At room temperature, the CuAAC reaction between [c2]daisy chain **3** and [2]semi-rotaxane **4** successfully afforded the formation of a white solid, hetero[4]rotaxane **5**, with a yield exceeding 50% in the presence of Cu(MeCN)_4_PF_6_. As shown in Fig. 4a and b, the terminal alkynyl proton H_11_ of **3** disappeared after the CuAAC click reaction, indicating the successful formation of the triazole. The resonance for the triazole proton H_f_ appeared at *δ* = 7.72 ppm and the adjacent protons H_10_, H_b_, H_d_ and H_e_ shifted downfield with a Δ*δ* of 0.38, 0.22, 0.12 and 0.89 ppm, respectively. All of these changes demonstrated the importance of the CuAAC click reaction for the formation of hetero[4]rotaxane **5**, as illustrated in Scheme 1.^15^ The high resolution mass spectrum (HRMS) of hetero[4]rotaxane **5** showed major signals at *m*/*z* 628.0955, 885.7819 and 1401.1543, which correspond to the product after the loss of 4, 3 and 2PF_6_^–^ ions, *i.e.* [M – 4PF_6_]^4+^, [M – 3PF_6_]^3+^ and, [M – 2PF_6_]^2+^, respectively. The molecular ion peaks of [M – 4PF_6_]^4+^ and [M – 3PF_6_]^3+^, as well as the results of ^1^H NMR, give enough evidence to show the formation of hetero[4]rotaxane **5**.^15^

**Scheme 2:**
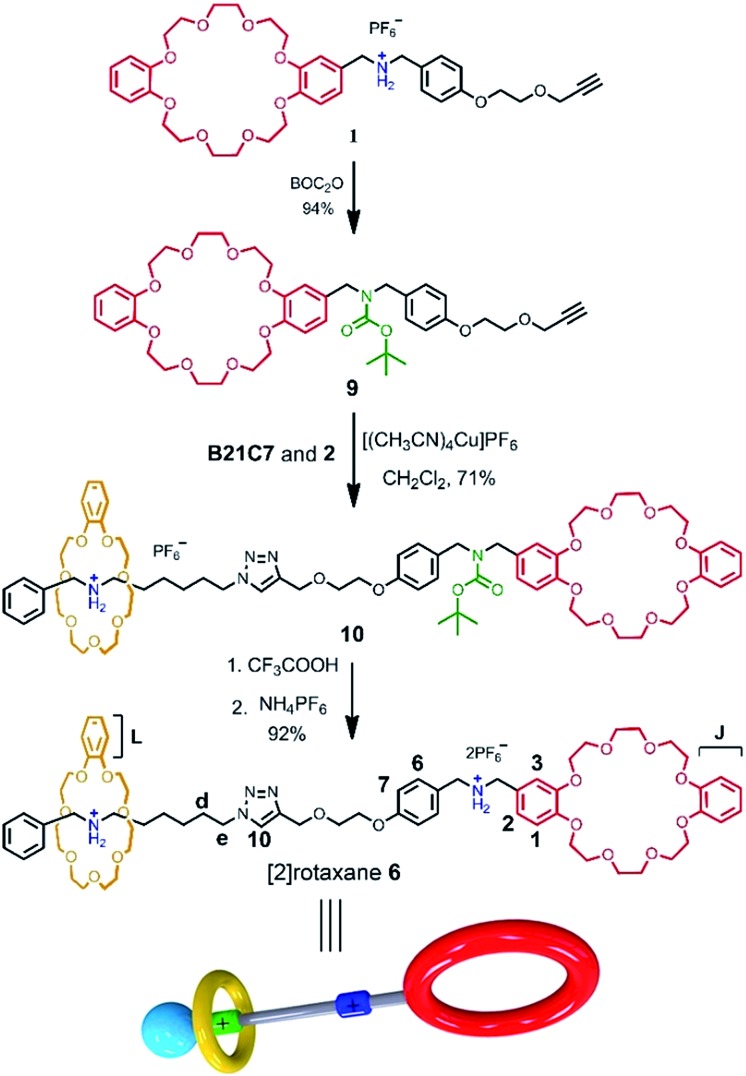
Synthetic route for [2]rotaxane **6**.

For further understanding of the self-sorting process and confirming the chemical structure of hetero[4]rotaxane **5**, the dibenzylammonium site in compound **1** was protected with a BOC group to yield compound **9** (Scheme 2), leading to a recognition failure between macrocyclic **DB24C8** and the DBA site. As a result, there was only one type of secondary ammonium recognition site in the mixed systems of compound **9**, compound **2** and **B21C7**, meaning that a non-self-sorting process would occur among these building blocks. Then, a CuAAC click reaction of compound **9** and [2]semi-rotaxane **4** successfully afforded [2]rotaxane **10** in the presence of Cu(MeCN)_4_PF_6_ in a 1 : 1 volume ratio of dichloromethane/acetonitrile at room temperature. After being treated with trifluoroacetic acid, the reference compound [2]rotaxane **6** with an unoccupied **DB24C8** ring was obtained. The HRMS spectrum of [2]rotaxane **6** shows major signals at *m*/*z* 1400.6526, corresponding to the product after a loss of the PF_6_^–^ ion, confirming the interlocked structure. Furthermore, we compared all of the resonances between hetero[4]rotaxane **5** and [2]rotaxane **6** by analysing their ^1^H NMR spectra (Fig. 4b and c). As shown in the ^1^H NMR spectra of **5** and **6** in [D_3_] acetonitrile (Fig. 4b and c), the protons H_1_, H_2_, H_3_ and H_J_ of [2]rotaxane **6** shifted downfield with a Δ*δ* of about 0.2 ppm, and the stereoisomer signals of H_1a_ and H_3b_ disappeared, which was attributed to the fact that the DBA site was not surrounded by the **DB24C8** macrocycle to give a simple chemical shift environment. It is notable that the DBA protons in [2]rotaxane **6** cannot be detected because there are no hydrogen bonding interactions between the crown ether and the DBA hydrogen atoms.^16^ Compared with the ^1^H NMR spectrum of hetero[4]rotaxane **5**, H_4_ and H_5_ which are adjacent to the DBA site were observed to shift upfield with a Δ*δ* of –0.53 ppm in the ^1^H NMR spectrum of [2]rotaxane **6**, which confirmed that the DBA sites were not bound by the host macrocycle **DB24C8**. Therefore, the phenyl-stopped **B21C7** played an important role in inhibiting the dethreading of the daisy chain structure in the middle, this is also called a cascade-stopped strategy.^10,7b^ Importantly, this inhibiting behaviour also caused the stable structure of hetero[4]rotaxane **5** instead of a metastable pseudorotaxane.

Meanwhile, 2D NOESY spectra of hetero[4]rotaxane **5** and [2]rotaxane **6** validated their chemical structures. The NOESY spectrum of **6** in [D_3_]acetonitrile (Fig. S1†) shows two cross-peaks (peak F and G) between the phenyl protons H_L_ and ethylene protons of **B21C7**, thus clearly indicating that **B21C7** is located on the outer ammonium site which is far away from the **DB24C8** ring. In the meantime, no cross-peaks between **DB24C8** and the inner DBA site were found. Nevertheless, in the NOESY spectrum of **5** (Fig. S2†), we found not only cross-peaks (peaks P, Q, R and S) between the phenyl protons H_L_ and ethylene protons of **B21C7**, but also cross-peaks (peak N and O) between the phenyl protons H_7_ and ethylene protons of **DB24C8**, and cross-peaks (peak T and U) between the phenyl protons H_6_ and ethylene protons of **DB24C8**, thus clearly indicating the existence of a [c2]daisy chain structure in **5**.

## Conclusion

In conclusion, a novel hetero[4]rotaxane containing a [c2]daisy chain can be facilely synthesized in one pot by employing a multi-component self-sorting strategy followed by the well-known CuAAC stoppering reaction. The highly selective self-assembling process that occurred among the three kinds of building blocks provides the possibility for the formation of only two kinds of rotaxane precursors, by employing a steric hindrance-related “language”. This work serves as one of few successful examples of the construction of hetero[*n*]rotaxane with a [c2]daisy chain cored structure directed by a self-sorting strategy. Significantly, the integration of hetero[*n*]rotaxane and a [c2]daisy chain structure enlarges the family of mechanically interlocked molecules and this synthetic methodology can be utilized in the preparation of mechanically interlocked compounds with increasingly complicated structures and functions.
