# Supplementary material for: One-pot synthesis of a [c2]daisy-chain-containing hetero[4]rotaxane via a self-sorting strategy
Source: Chem Sci. 2016 Jan 12;7(3):1696–701. doi: 10.1039/c5sc04844c (PMC5535066; doi:10.1039/c5sc04844c)
Supplement: Supplementary file 1 [file SC-007-C5SC04844C-s001.pdf]

Electronic Supplementary Material (ESI) for Chemical Science.
This journal is © The Royal Society of Chemistry 2016

SUPPORTING INFORMATION

One-pot Synthesis of a [c2]Daisy-Chain-Containing Hetero[4]rotaxane *via* a Self-sorting Strategy

Xin Fu, Qi Zhang, Si-Jia Rao, Da-Hui Qu* and He Tian

Key Laboratory for Advanced Materials and Institute of Fine Chemicals, East China University of Science and Technology, 130 Meilong Road, Shanghai, 200237 (China).

1. General Information

Chemicals were purchased from Adamas-beta®, Aldrich or TCI and used as received unless otherwise stated. Solvents were reagent grade, which were dried and distilled prior to use according to standard procedures. All reactions were carried out under an atmosphere of dry nitrogen unless otherwise stated. The molecular structures were confirmed using ^{1}H NMR, ^{13}C NMR, and high resolution ESI mass spectroscopy. NMR experiments (^{1}H NMR, ^{13}C NMR and NOESY spectra) were measured on a Brüker AV-400 spectrometer (^{1}H, 400 MHz; ^{13}C, 100 MHz). The electronic spray ionization (ESI) mass spectra were tested on a LCT Premier XE mass spectrometer. Compound **8** has been synthesized according to the procedure described by S. J. Cantrill, G. J. Youn, J. F. Stoddart[S1] as a white solid. Compound **7** was synthesized according to our previously published methods.[S2]

2. Synthesis

Preparation of 1: (2-Formyl)dibenzo[24]crown-8 **8** (1.0 g, 2.1 mmol) and compound **7** (0.48 g, 2.5 mmol) were dissolved in dry methanol (30 mL) and the mixture was heated under reflux in an argon atmosphere for 30 hours. After cooling, $NaBH_4$ (0.40 g, 10.5 mmol) was added by portions at 0 °C. The mixture was stirred at room temperature for 5 h; then, 20 ml water was added to the mixture slowly to quench the reaction, Methanol was evaporated, and the residue was extracted with CH_2Cl_2 (3 × 20mL). The combined organic phase was washed with H_2O (2 × 20mL). The organic layer was dried over Na_2SO_4 and concentrated under reduced pressure. The residue was diluted with acetone (50 mL) and HCl (2 mL) was added. The mixture was stirred for 1h and concentrated under reduced pressure to give a colourless oil, the residue was dissolved in acetone (8 mL) quickly and cooled at 0 °C, yielding a white precipitate slowly. The precipitate was collected and washed with acetone (5 mL). To a solution of the previous solid in water (50 mL) was added methanol dropwise until all the solid was dissolved, another 5 ml of methanol was added. Then, saturated aqueous NH_4PF_6 solution (10 mL) was added, a white precipitate appeared quickly, the mixture was

stirred vigorously for 20 min. The reaction mixture was filtered, the precipitate was collected and washed with H_2O to give the product **1** (1.32g, 78%) as a white powder. ^{1}H NMR (400 MHz, DMSO, 298 K): δ 8.96, (s, 2H), 7.39 (d, *J* = 8.8 Hz, 2H), 7.09 (s, 1H), 7.03-6.97 (m, 4H), 6.96-6.91 (m, 2H), 6.90-6.85 (m, 2H). 4.21 (d, *J* = 2.4 Hz, 2H), 4.15-4.11 (m, 2H), 4.10-4.00 (m, 12H), 3.82-3.73 (m, 10H), 3.66 (d, *J* = 2.8 Hz, 8H), 3.48 (t, *J* = 2.4 Hz, 1H). ^{13}C NMR (100 MHz, DMSO, 298 K): δ 158.90, 148.90, 148.41, 148.17, 131.54, 124.16, 123.74, 122.95, 121.13, 115.38, 114.53, 113.99, 113.95, 113.43, 80.14, 77.33, 70.43, 70.40, 69.14, 69.02, 68.81, 68.72, 68.65, 67.62, 66.89, 57.59, 49.67, 49.31. HRMS (ESI) (m/z): $[M - PF_6]^+$ calcd for $C_{37}H_{48}NO_{10}$ 666.3278, found 666.3275.

One-Pot Synthesis of Hetero[4]rotaxane 5: In a typical procedure, to a solution of compound **1** (500 mg, 0.62 mmol), compound **2** (280 mg, 0.74 mmol) and B21C7 (329 mg, 0.93 mmol) in CH_2Cl_2/CH_3CN (10.0 mL/10.0 mL), $Cu(CH_3CN)_4PF_6$ (347 mg, 0.93 mmol) was added. The mixture was stirred at room temperature protected by argon atmosphere for 3 days. Then, 20.0 mL CH_2Cl_2 was added, the mixture was washed with brine (3 × 20 mL), and dried over Na_2SO_4. After removal of the solvent, the crude product was purified by chromatography on a silica gel column (CH_2Cl_2/Methanol = 100/1) to give hetero[4]rotaxane **5** (495 mg, 52%) as a white solid. ^{1}H NMR (400 MHz, CD_3CN, 298 K): δ 7.72 (s, 2H), 7.55 (s, 4H), 7.47 (d, *J* = 8.8 Hz, 4H), 7.40-7.33 (m, 10H), 7.30 (s, 2H), 7.09 (s, 2H), 7.00-6.90 (m, 13H), 6.88 (d, J = 8.4 Hz, ~0.5H), 6.86-6.74 (m, ~10.5H), 6.40 (d, *J* = 8.4 Hz, ~1.5H), 6.15 (s, ~0.5H), 4.78-4.49 (m, 12H), 4.39-4.33 (m, 4H), 4.31-4.08 (m, 24H), 4.07-3.42 (m, 80H), 3.39-3.32 (m, 8H), 1.82-1.74 (m, 4H), 1.56-1.47 (m, 4H), 1.33-1.26 (m, 4H), 1.23-1.14 (m, 4H). 13C NMR (100 MHz, CD_3CN, 298 K): δ 160.41, 148.77, 147.92, 147.30, 147.08, 133.91, 131.78, 131.71, 131.17, 131.07, 130.10, 130.04, 129.72, 125.89, 125.48, 123.74, 122.25, 121.77, 115.79, 114.40, 112.98, 112.92, 112.89, 72.76, 72.61, 72.14, 71.99, 71.60, 71.43, 71.37, 70.78, 70.64, 69.47, 69.23, 68.74, 68.48, 68.36, 68.12, 64.90, 53.03, 52.68, 51.73, 50.89, 47.90, 30.62, 27.04, 26.98, 26.52, 23.21. HRMS (ESI) (m/z): $[M - 2PF_6]^{2+}$ calcd for

$C_{136}H_{194}F_{12}N_{10}O_{34}P_2/2$ 1401.1538, found 1401.1543; [M - $3PF_6]^{3+}$ calcd for $C_{136}H_{194}F_6N_{10}O_{34}P/3$ 885.7811, found 885.7819; [M - $4PF_6]^{4+}$ calcd for $C_{136}H_{194}N_{10}O_{34}/4$ 628.0948, found 628.0955.

Preparation of 9: A mixture of compound **1** (1.00 g, 1.23 mmol), di-tert-butyl dicarbonate (1.34 g, 6.15 mmol), and triethylamine (1.0 mL) was stirred in dry CH_2Cl_2 (30.0 mL) at room temperature under argon for 12 h. Then, the mixture was washed with brine (3 × 20 mL), and dried over Na_2SO_4. After removal of the solvent, the crude product was purified by chromatography on a silica gel column (CH_2Cl_2/Methanol = 100/1) to give compound **9** (887 mg, 94%) as a white solid. ^1H NMR (400 MHz, CD_3CN, 298 K): δ 7.15 (d, *J* = 8.0 Hz, 2H), 6.95-6.84 (m, 7H), 6.74 (d, *J* = 8.0 Hz, 2H), 4.26 (s, 4H), 4.21 (d, *J* = 2.4 Hz, 2H), 4.12-4.06 (m, 8H), 4.04-4.00 (m, 2H), 3.82-3.75 (m, 10H), 3.70-3.64 (m, 8H), 2.73 (t, *J* = 2.4 Hz, 1H), 1.44 (s, 9H). ^{13}C NMR (100 MHz, CD_3CN, 298 K): δ 158.95, 156.60, 149.80, 149.69, 148.82, 131.83, 130.07, 122.30, 121.38, 115.35, 115.01, 114.79, 80.76, 80.39, 75.90, 71.56, 70.47, 70.39, 69.88, 69.74, 69.68, 69.11, 68.14, 58.84, 28.64. HRMS (ESI) (m/z): $[M + Na]^+$ calcd for $C_{42}H_{55}NO_{12}Na$ 788.3622, found 788.3621.

Preparation of 10: To a solution of compound **9** (200 mg, 0.26 mmol), compound **2** (148 mg, 0.39 mmol) and B21C7 (185 mg, 0.52 mmol) in dry CH_2Cl_2 (10.0 mL), $Cu(CH_3CN)_4PF_6$ (194 mg, 0.52 mmol) was added. The mixture was stirred at room temperature in a argon atmosphere for 3 days. Then, 20.0 mL CH_2Cl_2 was added, the mixture was washed with brine (3 × 20 mL), and dried over Na_2SO_4. After removal of the solvent, the crude product was purified by chromatography on a silica gel column (CH_2Cl_2/methanol = 100/1) to give compound **10** (278 mg, 71%) as a white solid. ^1H NMR (400 MHz, CD_3CN, 298 K): δ 7.74 (s, 1H), 7.55 (s, 2H), 7.40-7.33 (m, 5H), 7.17 (d, *J* = 8.4 Hz, 2H), 7.14-7.10 (m, 2H), 7.08-7.02 (m, 3H), 6.99-6.84 (m, 8H), 4.60 (s, 2H), 4.39-4.14 (m, 20H), 4.11-4.07 (m, 2H), 3.88-3.80 (m, 4H), 3.79-3.70 (m, 10H), 3.68-3.49 (m, 18H), 3.47-3.39 (m, 4H), 3.38-3.32 (m, 4H), 1.82-1.74 (m, 2H), 1.56-1.48 (m, 2H), 1.46 (s, 9H), 1.33-1.26 (m, 2H), 1.23-1.14 (m, 2H). ^{13}C NMR (100 MHz, CD_3CN, 298 K): δ 159.02, 156.59, 149.25, 149.12, 149.09, 148.08, 147.93, 133.92, 131.78, 131.18, 130.10, 129.71, 124.17, 124.10, 123.13, 122.26, 117.67, 117.48,

117.43, 116.70, 115.35, 112.99, 80.58, 72.13, 71.98, 71.60, 71.36, 70.63, 69.76, 69.63, 69.59, 69.32, 69.23, 68.60, 68.48, 68.34, 68.15, 68.12, 68.08, 68.01, 65.00, 51.74, 50.65, 47.91, 30.68, 28.65, 27.05, 26.98, 26.53. HRMS (ESI) (m/z): $[M - PF_6]^+$ calcd for $C_{73}H_{104}N_5O_{19}$ 1354.7326, found 1354.7321.

Synthesis of [2]rotaxane 6: A mixture of compound **10** (100 mg, 0.067 mmol) and trifluoroacetic acid (0.1 mL, 1.33 mmol) was stirred in CH_2Cl_2 (5.0 mL) at room temperature under argon for 2 h. After the solvent was reduced under vacuum, the residue was dissolved with CH_2Cl_2 (5.0 mL). Then, saturated aqueous NH_4PF_6 solution (2.0 mL) was added, the mixture was stirred for 3 h and washed with brine (3 × 20 mL). After removal of the solvent, the crude product was purified by chromatography on a silica gel column (CH_2Cl_2/Methanol = 100/1) to give [2]Rotaxane **6** (95 mg, 92%) as a white solid. ^1H NMR (400 MHz, CD_3CN, 298 K): δ 7.72 (s, 1H), 7.56 (s, 2H), 7.40-7.32 (m, 7H), 7.18 (s, 1H), 7.16-7.09 (m, 4H), 7.08-7.03 (m, 2H), 6.99-6.91 (m, 6H), 4.60 (s, 2H), 4.39-4.34 (m, 2H), 4.31-4.16 (m, 14H), 4.15-4.11 (m, 2H), 4.10-4.05 (m, 4H), 3.89-3.81 (m, 4H), 3.79-3.71 (m, 10H), 3.69-3.64 (m, 2H), 3.63-3.49 (m, 16H), 3.48-3.41 (m, 4H), 3.39-3.32 (m, 4H), 1.82-1.74 (m, 2H), 1.56-1.48 (m, 2H), 1.33-1.26 (m, 2H), 1.23-1.14 (m, 2H). ^{13}C NMR (100 MHz, CD_3CN, 298 K): δ 160.56, 149.93, 149.27, 149.07, 149.05, 147.96, 145.32, 133.95, 132.54, 131.19, 130.11, 129.72, 125.70, 124.24, 124.13, 123.99, 122.28, 118.90, 117.52, 117.12, 115.77, 113.02, 72.15, 72.01, 71.63, 71.40, 70.67, 69.79, 69.69, 69.47, 69.30, 69.26, 69.21, 68.87, 68.83, 68.65, 68.63, 68.49, 68.33, 68.25, 68.16, 64.98, 52.01, 51.86, 51.77, 50.68, 47.96, 30.69, 27.08, 27.00, 26.55. HRMS (ESI) (m/z): $[M - PF_6]^+$ calcd for $C_{68}H_{97}F_6N_5O_{17}P$ 1400.6521, found 1400.6526.

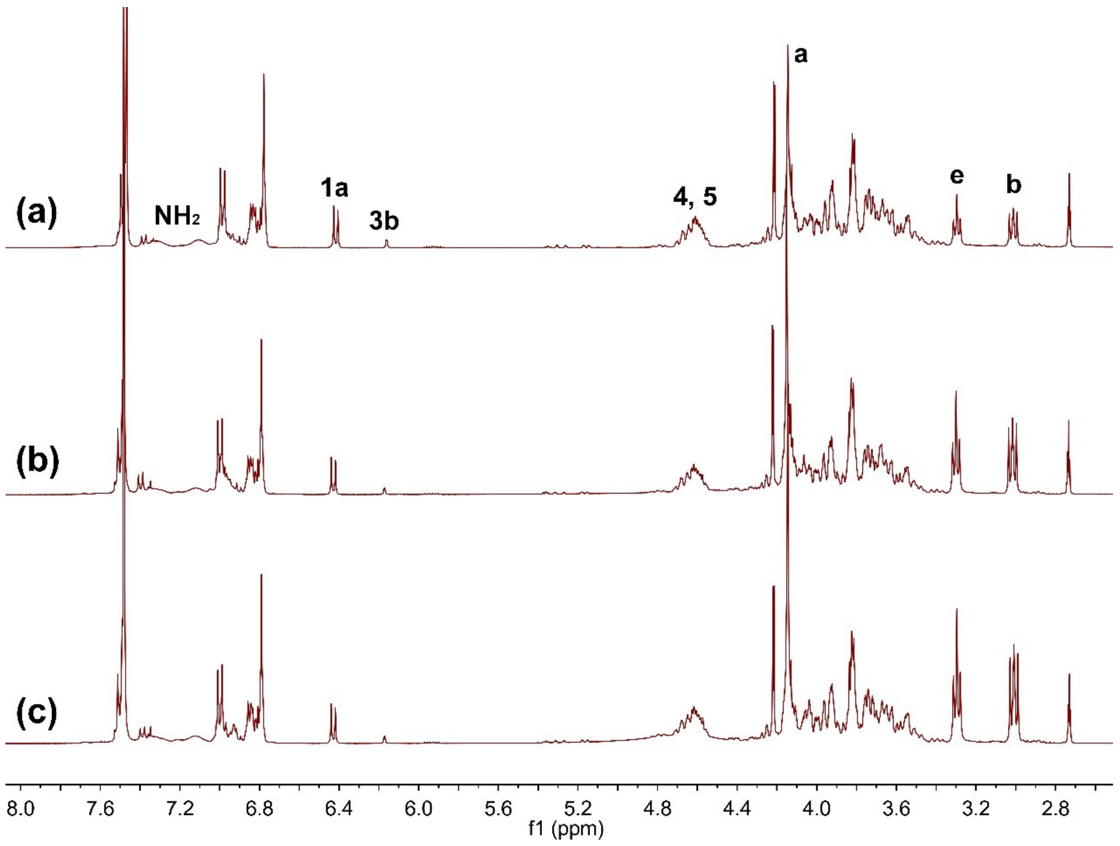

Figure S1. Partial ^{1}H NMR spectra (400 MHz, 298 K, [D3]acetonitrile) of (a) 2:1; (b) 1:1; (c) 1:2 mixture of **1** and **2**.

3. NOESY spectra

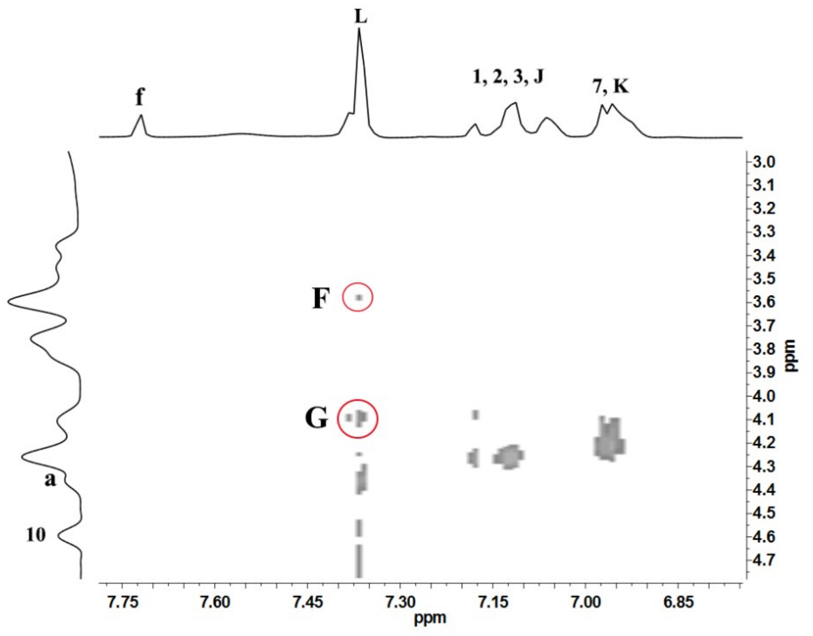

Figure S2. Partial NOESY spectrum (400 MHz, 298 K, [D$_3$]acetonitrile) of [2]rotaxane **6**.

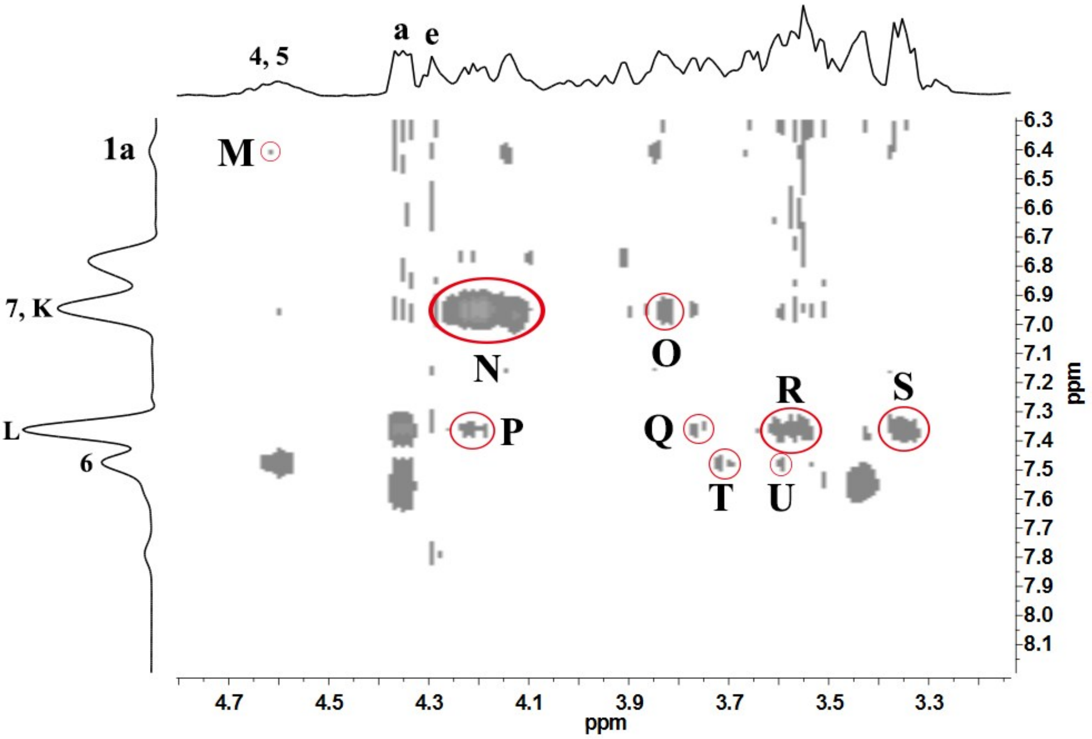

Figure S2. Partial NOESY spectrum (400 MHz, 298 K, [D$_3$]acetonitrile) of hetero[4]rotaxane **5**.

Supporting References:

[S1] S. J.Cantrill, G. J. Youn, J. F. Stoddart, D. J. Williams, *J. Org. Chem.* **2001**, *66*, 6857-6872.

[S2] H. Li, H. Zhang, Q. Zhang, Q.-W. Zhang, D.-H. Qu, *Org. Lett.* **2012**, *14*, 5900-5903.

Additional Spectra

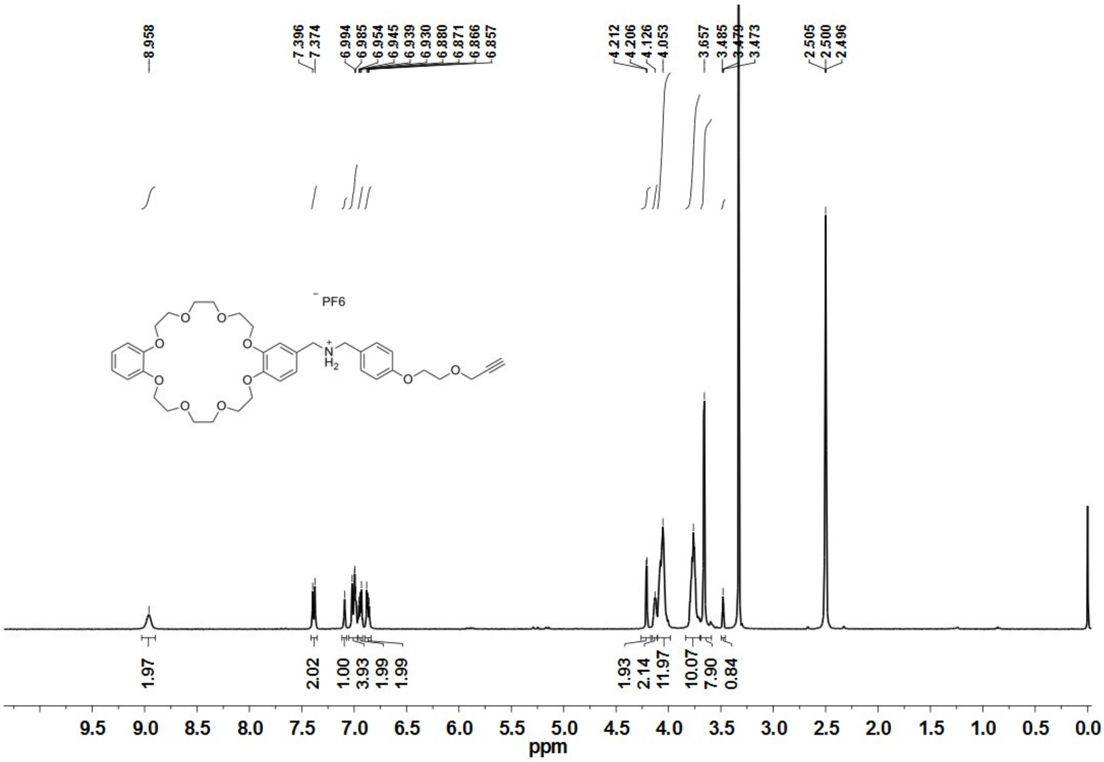

^{1}H NMR spectrum of **1** ([D_6]DMSO , 400MHz, 298K).

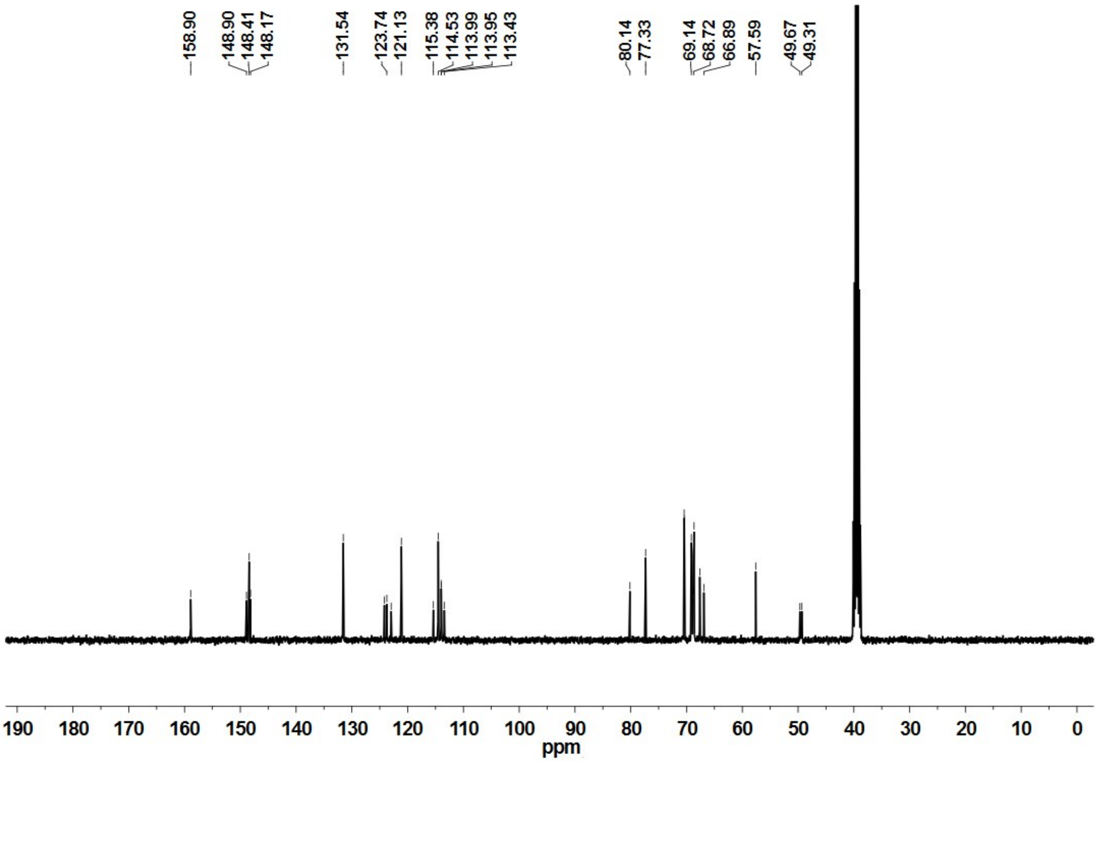

^{13}C NMR spectrum of **1** ([D$_6$]DMSO , 100MHz, 298K).

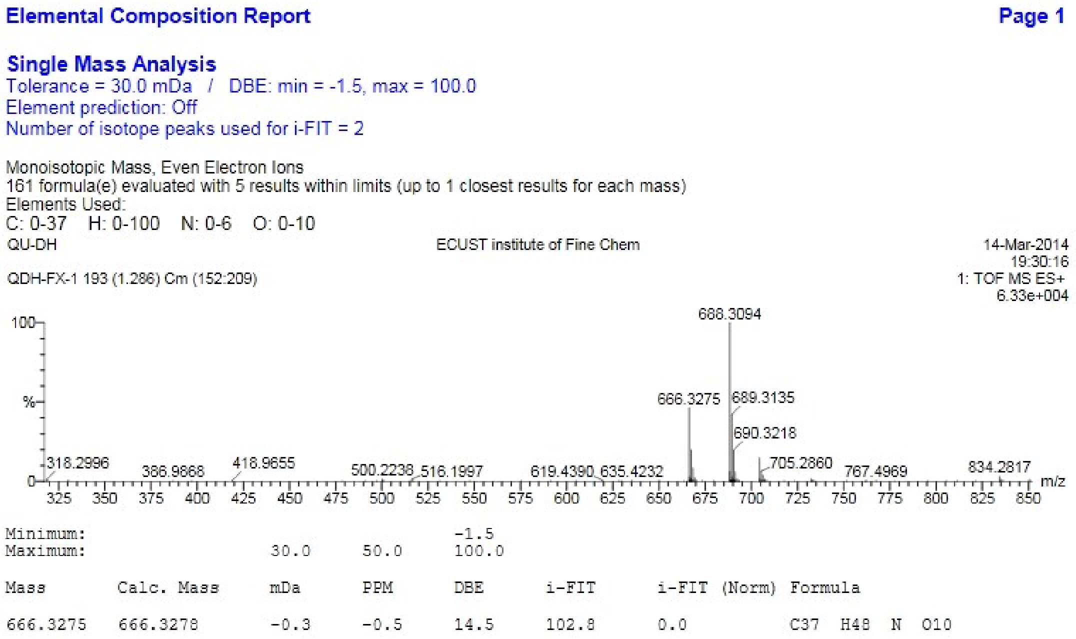
Elemental Composition Report Page 1

Single Mass Analysis
Tolerance = 30.0 mDa / DBE: min = -1.5, max = 100.0
Element prediction: Off
Number of isotope peaks used for i-FIT = 2

Monoisotopic Mass, Even Electron Ions
161 formula(e) evaluated with 5 results within limits (up to 1 closest results for each mass)
Elements Used:
C: 0-37 H: 0-100 N: 0-6 O: 0-10

QU-DH ECUST institute of Fine Chem 14-Mar-2014 19:30:16

QDH-FX-1 193 (1.286) Cm (152:209) 1: TOF MS ES+ 6.33e+004

Minimum:				-1.5			
Maximum:		30.0	50.0	100.0			
Mass	Calc. Mass	mDa	PPM	DBE	i-FIT	i-FIT (Norm)	Formula
666.3275	666.3278	-0.3	-0.5	14.5	102.8	0.0	C37 H48 N O10

ESI-Mass spectrum of **1**. [M - PF$_6$]$^+$ calcd for $C_{37}H_{48}NO_{10}$ 666.3278, found 666.3275.

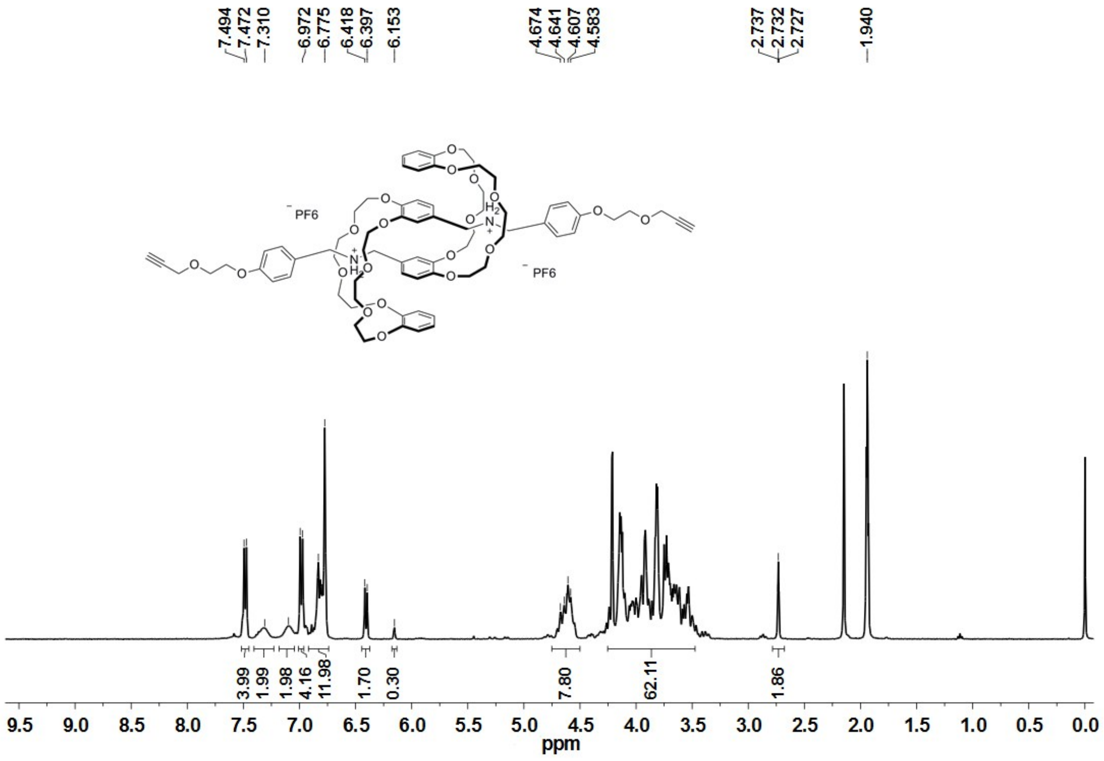

^{1}H NMR spectrum of [c2]daisy chain **3** ([D$_3$]acetonitrile , 400MHz, 298K).

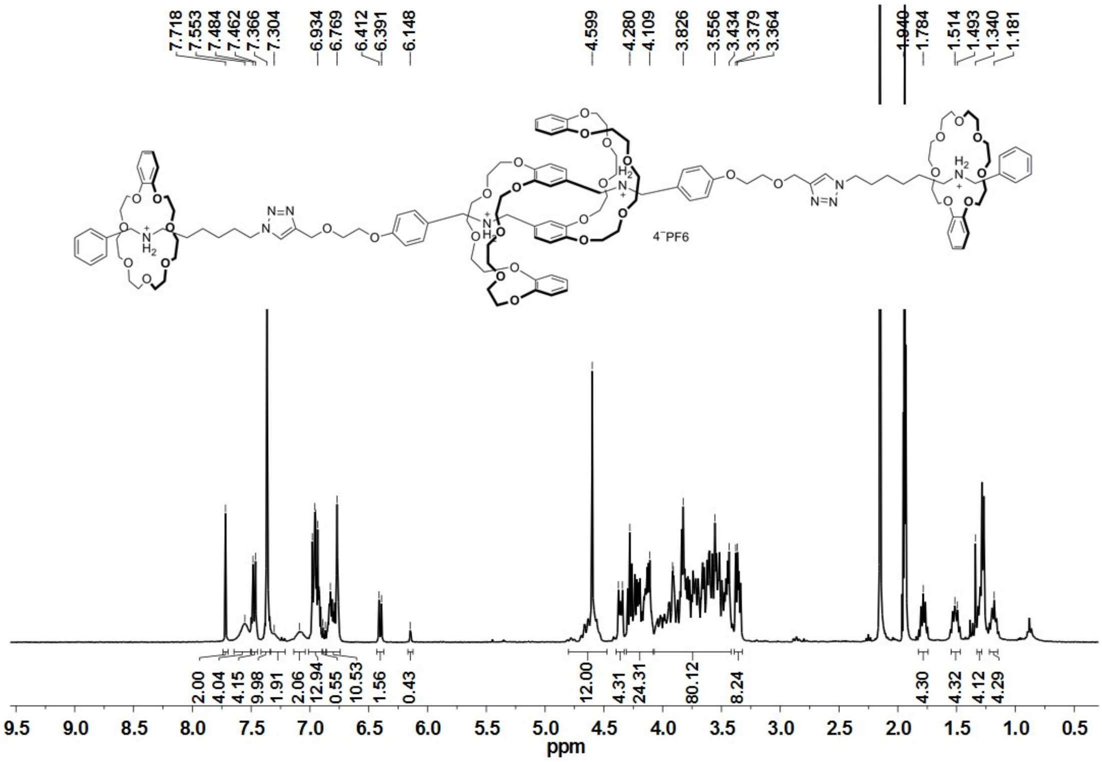

^{1}H NMR spectrum of hetero[4]rotaxane **5** ([D_3]acetonitrile , 400MHz, 298K).

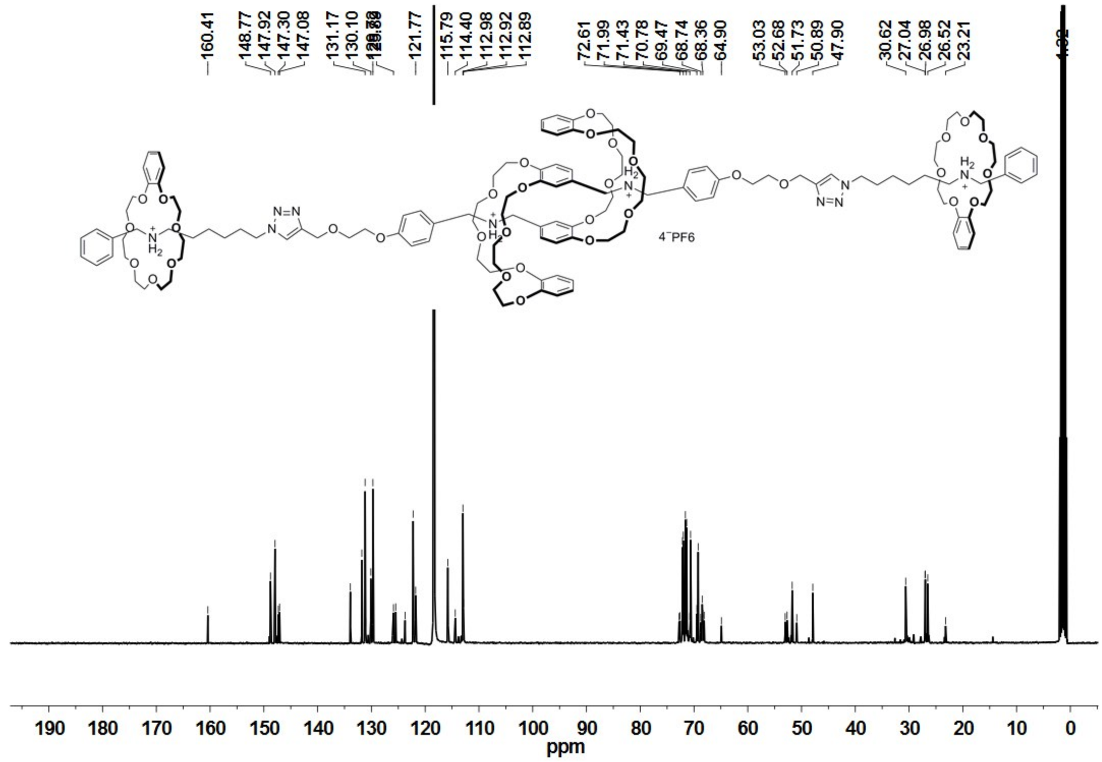

^{13}C NMR spectrum of hetero[4]rotaxane **5** ([D_3]acetonitrile , 100MHz, 298K).

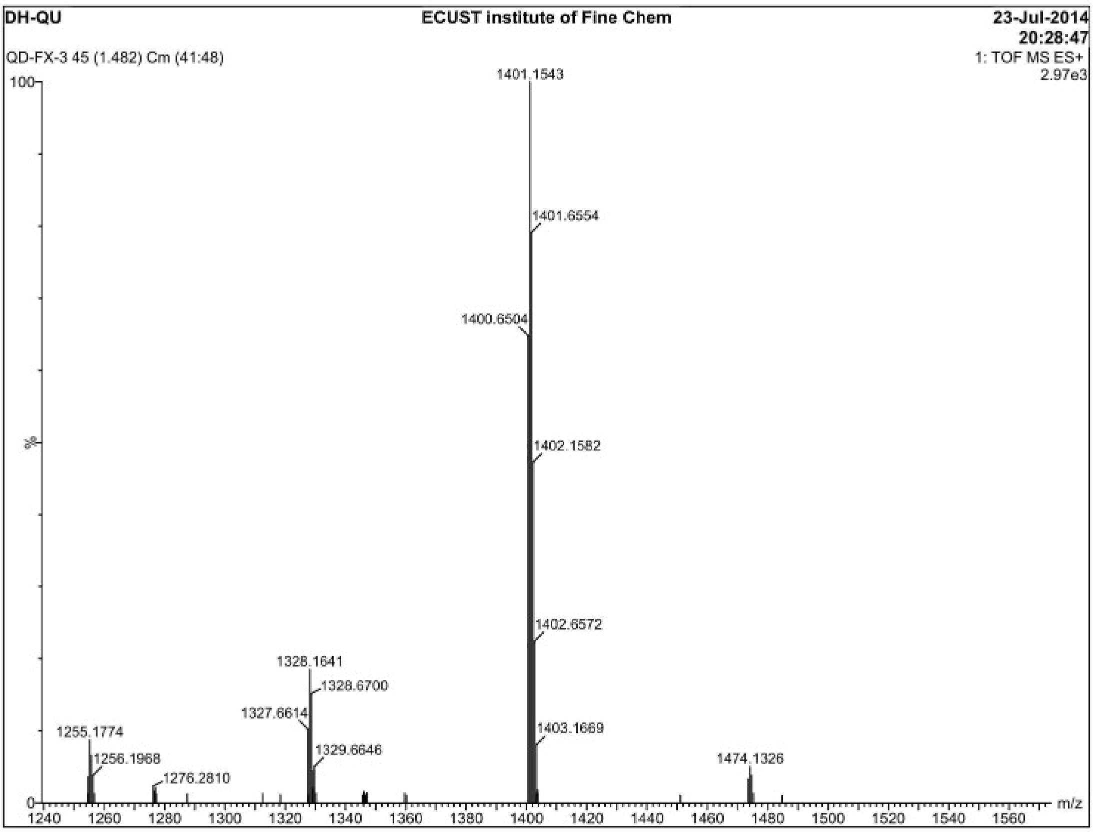

ESI-Mass spectrum of hetero[4]rotaxane **5** ([M - 2PF$_6$]$^{2+}$). [M - 2PF$_6$]$^{2+}$ calcd for $C_{136}H_{194}F_{12}N_{10}O_{34}P_2$/2 1401.1538, found 1401.1543.

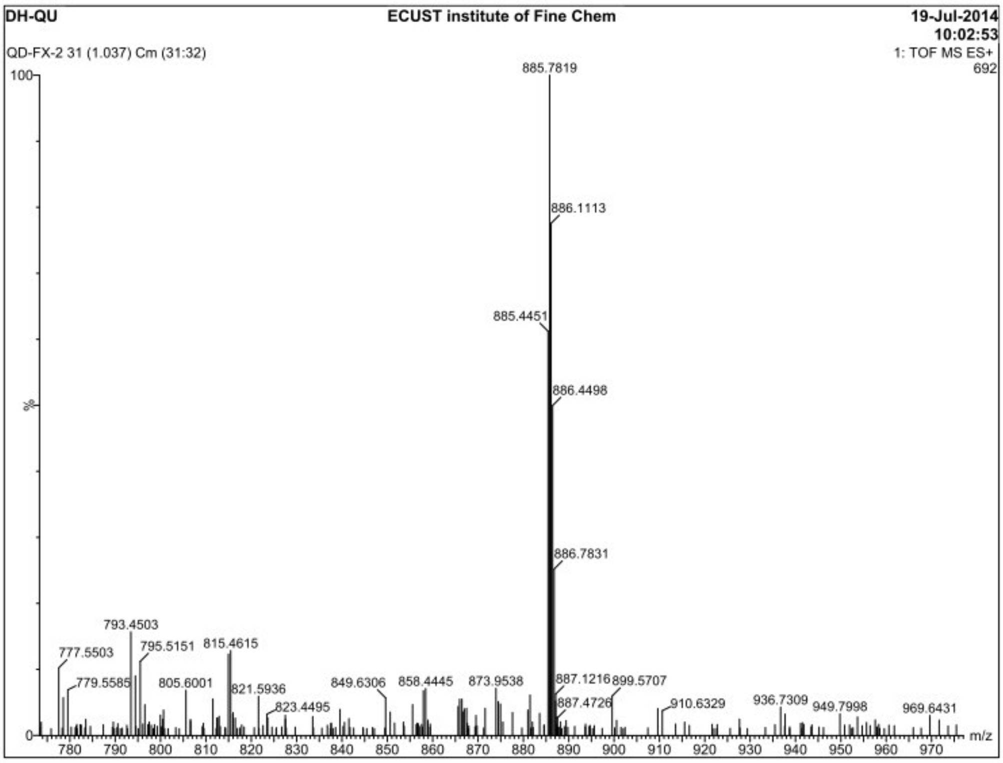

ESI-Mass spectrum of hetero[4]rotaxane **5** $[M - 3PF_6]^{3+}$ calcd for $C_{136}H_{194}F_6N_{10}O_{34}P/3$ 885.7811, found 885.7819.

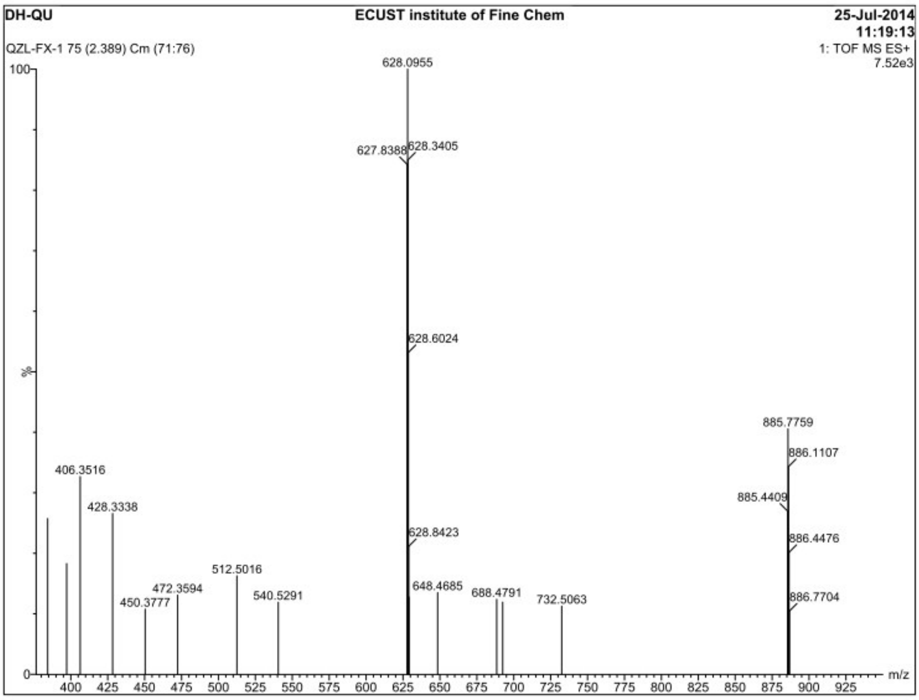

ESI-Mass spectrum of hetero[4]rotaxane **5** ($[M - 4PF_6]^{4+}$). $[M - 4PF_6]^{4+}$ calcd for $C_{136}H_{194}N_{10}O_{34}/4$ 628.0948, found 628.0955.

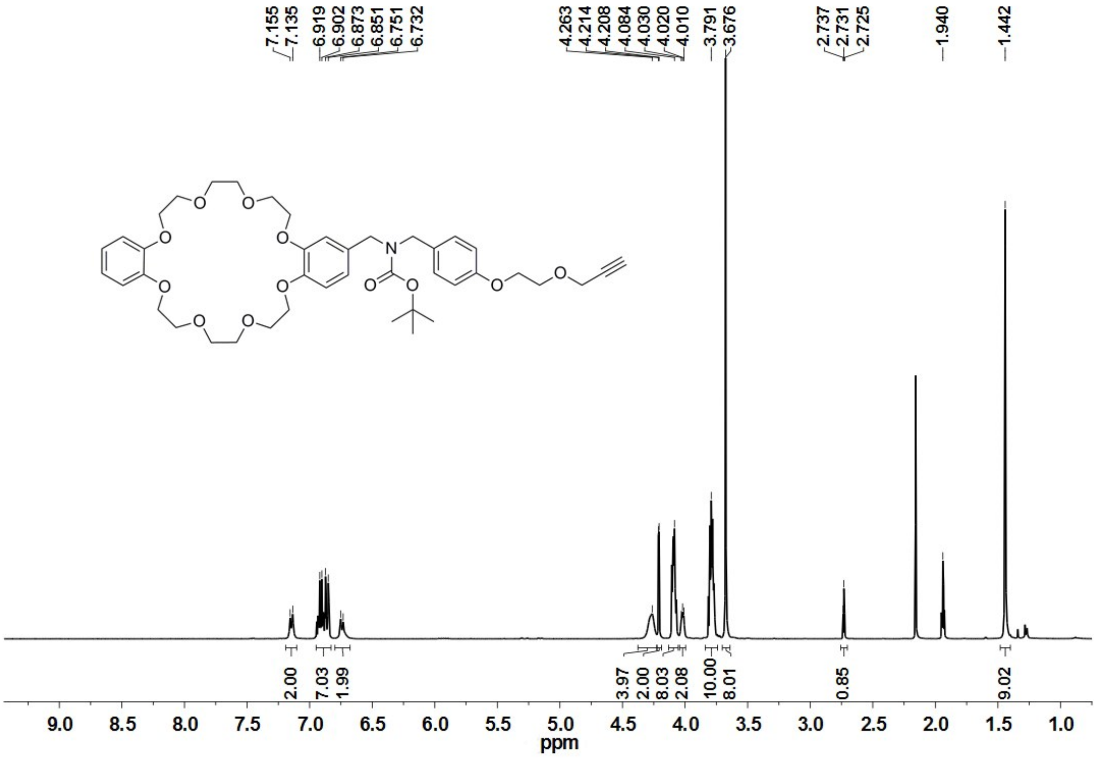

^{1}H NMR spectrum of **9** ([D_3]acetonitrile , 400MHz, 298K).

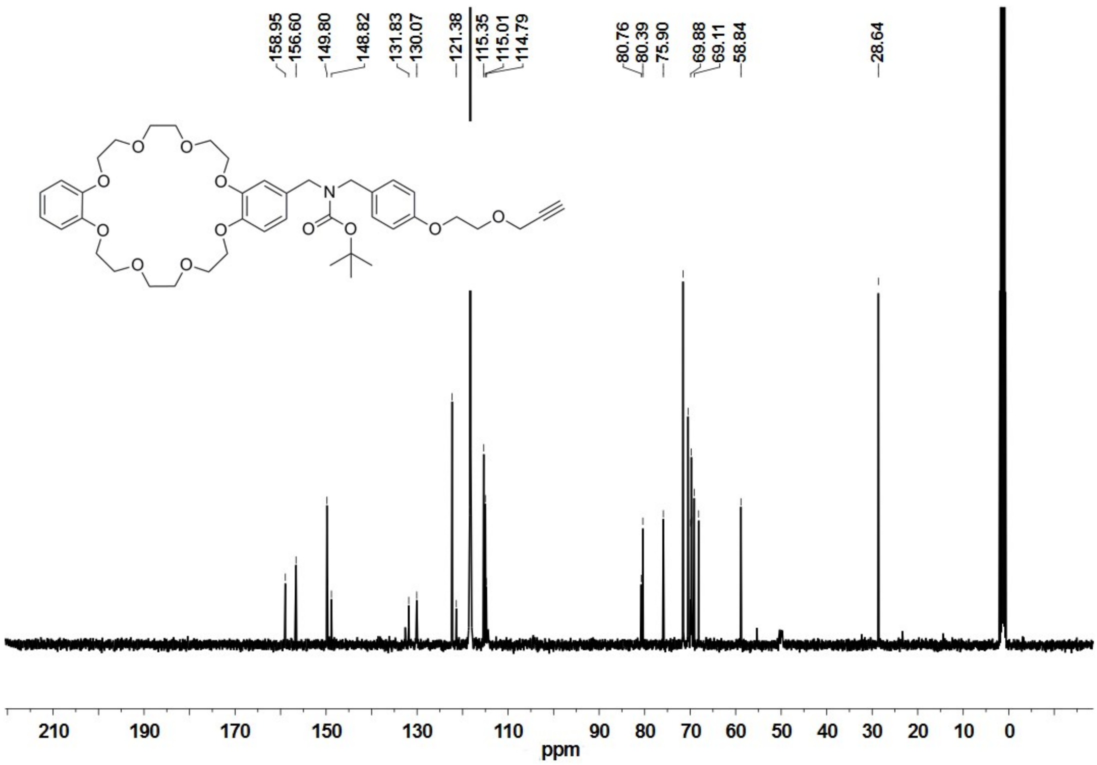

^{13}C NMR spectrum of **9** ([D_3]acetonitrile , 100MHz, 298K).

Elemental Composition Report **Page 1**

Single Mass Analysis
Tolerance = 30.0 mDa / DBE: min = -1.5, max = 100.0
Element prediction: Off
Number of isotope peaks used for i-FIT = 2

Monoisotopic Mass, Even Electron Ions
45 formula(e) evaluated with 1 results within limits (up to 1 best isotopic matches for each mass)
Elements Used:
C: 0-42 H: 0-56 N: 0-1 O: 0-12 Na: 0-1

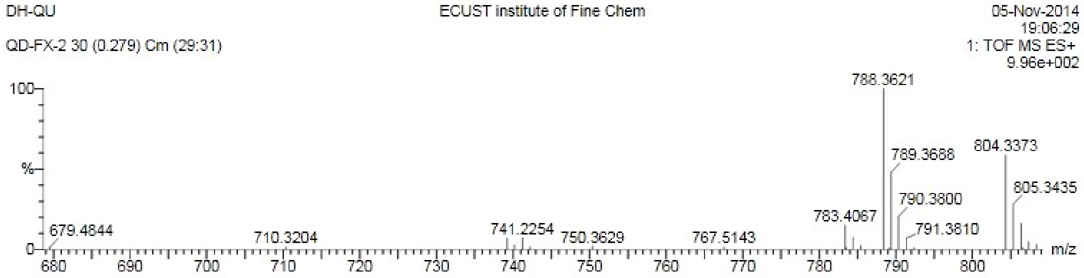

Minimum:				-1.5			
Maximum:		30.0	50.0	100.0			
Mass	Calc. Mass	mDa	PPM	DBE	i-FIT	i-FIT (Norm)	Formula
788.3621	788.3622	-0.1	-0.1	15.5	13.5	0.0	C42 H55 N O12 Na

ESI-Mass spectrum of **9**. $[M + Na]^+$ calcd for $C_{42}H_{55}NO_{12}Na$ 788.3622, found 788.3621.

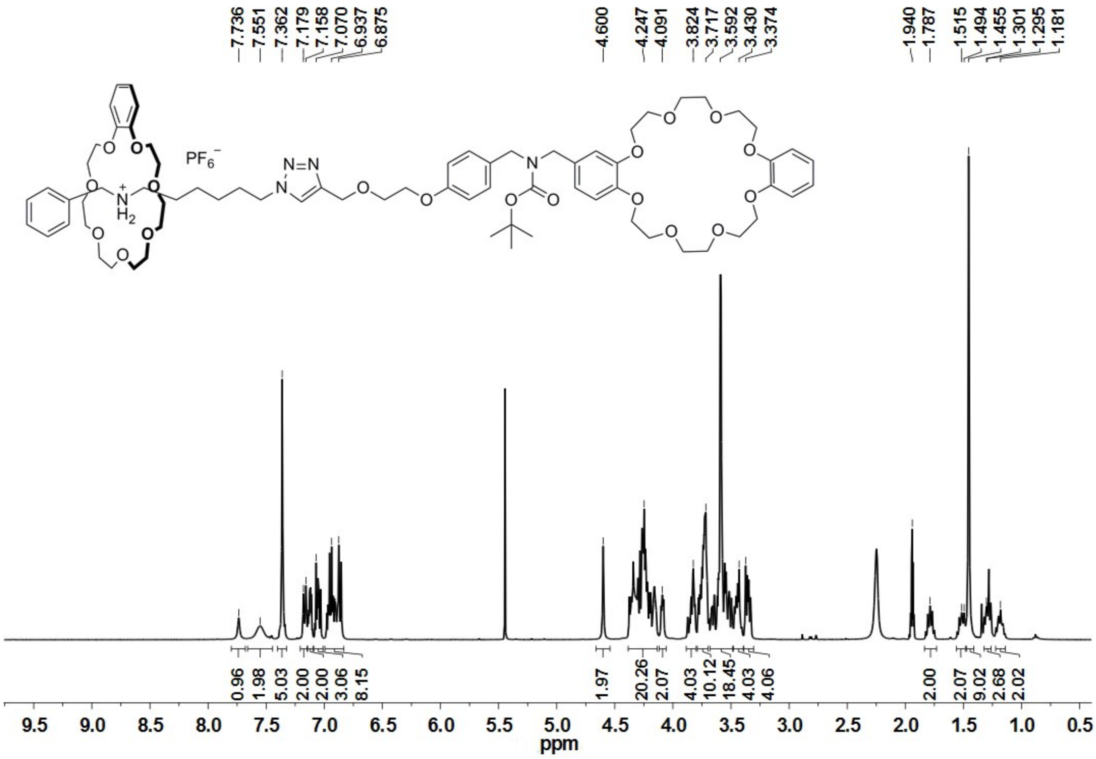

^{1}H NMR spectrum of **10** ($[D_3]$acetonitrile , 400MHz, 298K).

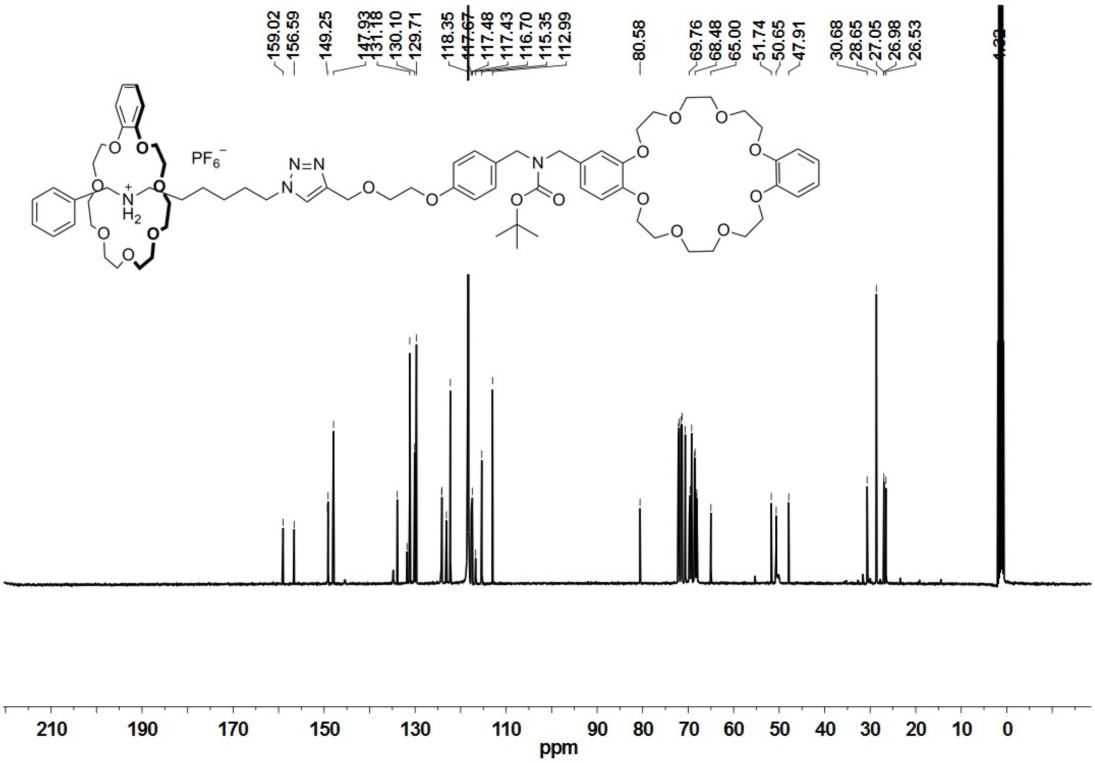

^{13}C NMR spectrum of **10** ([D$_3$]acetonitrile , 100MHz, 298K).

Elemental Composition Report Page 1

Single Mass Analysis
Tolerance = 30.0 mDa / DBE: min = -1.5, max = 100.0
Element prediction: Off
Number of isotope peaks used for i-FIT = 2

Monoisotopic Mass, Even Electron Ions
111 formula(e) evaluated with 1 results within limits (up to 1 best isotopic matches for each mass)
Elements Used:
C: 0-73 H: 0-105 N: 0-5 O: 0-19

QU-DH ECUST institute of Fine Chem 23-Jan-2015 16:53:00
QD-FX-23 38 (1.258) Cm (38:41) 1: TOF MS ES+ 3.57e+004

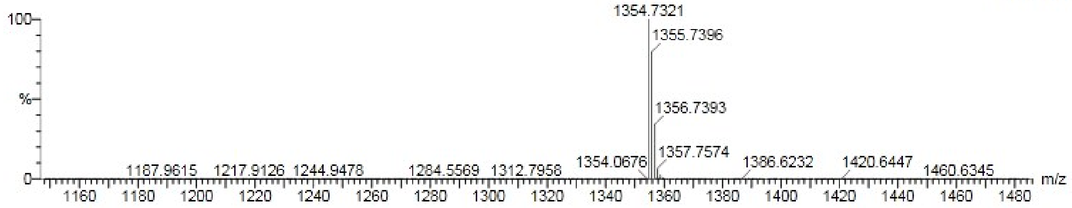

Minimum:				-1.5			
Maximum:		30.0	50.0	100.0			
Mass	Calc. Mass	mDa	PPM	DBE	i-FIT	i-FIT (Norm)	Formula
1354.7321	1354.7326	-0.5	-0.4	24.5	25.6	0.0	C73 H104 N5 O19

ESI-Mass spectrum of **10**. [M - PF$_6$]$^+$ calcd for $C_{73}H_{104}N_5O_{19}$ 1354.7326, found 1354.7321.

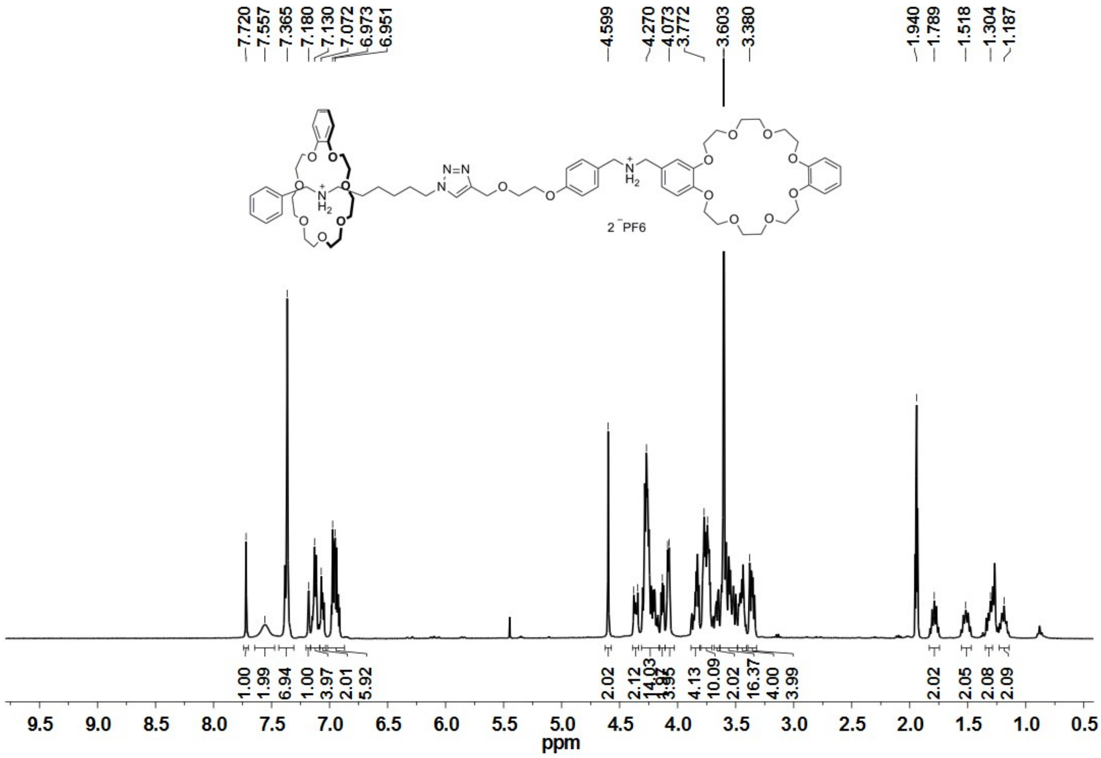

^{1}H NMR spectrum of [2]rotaxane **6** ([D_3]acetonitrile , 400MHz, 298K).

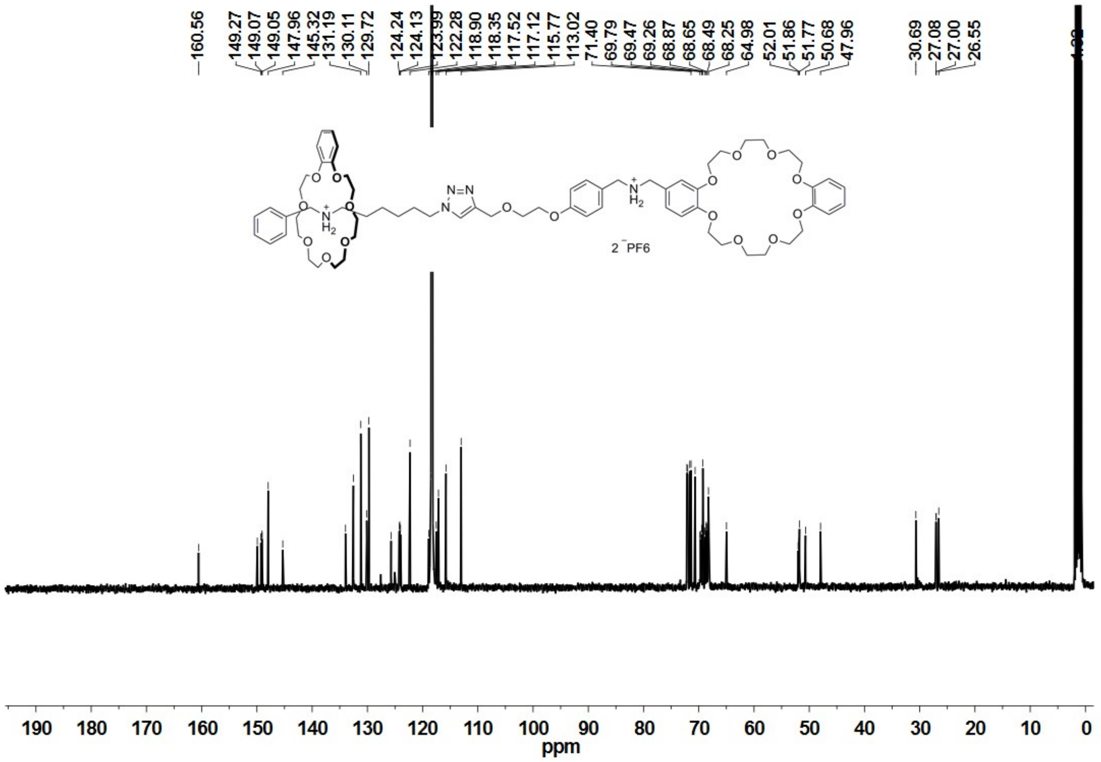

^{13}C NMR spectrum of [2]rotaxane **6** ([D_3]acetonitrile , 100MHz, 298K).

Elemental Composition Report

Single Mass Analysis
Tolerance = 30.0 mDa / DBE: min = -1.5, max = 100.0
Element prediction: Off
Number of isotope peaks used for i-FIT = 2

Monoisotopic Mass, Even Electron Ions
2211 formula(e) evaluated with 3 results within limits (up to 1 best isotopic matches for each mass)
Elements Used:
C: 0-68 H: 0-100 N: 0-5 O: 0-17 F: 0-6 P: 0-2

QU-DH ECUST institute of Fine Chem 23-Jan-2015 16:47:31

QD-FX-24 11 (0.422) Cm (7:11) 1: TOF MS ES+ 4.93e+003

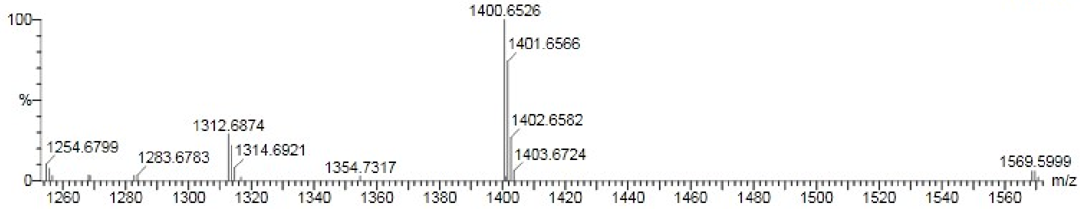

Mass	Calc. Mass	mDa	PPM	DBE	i-FIT	i-FIT (Norm)	Formula
Minimum:				-1.5			
Maximum:		30.0	50.0	100.0			
1400.6526	1400.6521	0.5	0.4	20.5	21.4	0.0	C68 H97 N5 O17 F6 P

ESI-Mass spectrum of [2]rotaxane **6**. $[M - PF_6]^+$ calcd for $C_{68}H_{97}F_6N_5O_{17}P$ 1400.6521, found 1400.6526.
